# Incidence and treatment-related risk factors of inhibitor development after intensive FVIII replacement for major orthopaedic surgery in previous treated haemophilia A

**DOI:** 10.1186/s13018-024-04843-4

**Published:** 2024-06-16

**Authors:** Han Wang, Wei Zhu, Shujie Wang, Bin Feng, Xisheng Weng

**Affiliations:** 1 Department of Orthopaedic Surgery, State Key Laboratory of Complex Severe and Rare Diseases, Peking Union Medical College Hospital, Chinese Academy of Medical Sciences & Peking Union Medical College, Shuai Fu Yuan 1#, Dongcheng District, Beijing, 100730 China; 2 Department of Hematology, Peking Union Medical College Hospital, Chinese Academy of Medical Sciences & Peking Union Medical College, Beijing, 100730 China

**Keywords:** Factor VIII, Previous treated haemophilia A, Major orthopaedic surgery, Inhibitor, Risk factors

## Abstract

**Introduction:**

Haemophilia A (HA) is an X-linked recessive bleeding disorder caused by lack or deficiency of coagulation factor VIII.

**Aim:**

The aim of this study is to determine the incidence and treatment-related risk factors of inhibitor development after intensive FVIII replacement for major orthopaedic surgery in previous treated persons with HA.

**Methods:**

A total of 151 HA who underwent 221 major orthopaedic surgical procedures after intensive FVIII treatment were reviewed. The results of inhibitor tests were collected. Potential clinical risk factors for inhibitor development were analyzed.

**Results:**

111 people were diagnosed with severe HA. Thirty-seven persons (24.5%) had history of previous intensive FVIII treatment for surgical procedure. They received a mean perioperative cumulative FVIII of 498 iu/kg within first week after surgery. Seven cases (4.6%) developed an inhibitor post-operatively in our study. Surgical procedure for pseudotumor and the group of persons who experienced postoperative complications had the higher incidence of inhibitor development (9.5%, 13.3% respectively). Only previous history for intensive FVIII exposure was considered as a significant predictor for postoperative inhibitor development after multivariate logistic regression analysis (*OR*: 29.5, *P* = 0.002).

**Conclusion:**

The incidence of inhibitor development in previously treated persons with HA undergoing major orthopaedic surgery was 4.6% and the history of previous intensive FVIII treatment for surgery was associated with higher risk of inhibitor development.

## Introduction

Haemophilia A(HA) is an X-linked recessive bleeding disorder caused by lack or deficiency of coagulation factor VIII (FVIII) which is encoded by *F8* gene, and often results in excessive bleeding and leads to musculoskeletal complications [1]. Over 90% of bleeding episodes in people with haemophilia A(PWHA) occur within the musculoskeletal system, which adds to their disability, and severely affect their quality of life [2]. Surgical treatment is effective to preserve and restore the function for the PWHA with end-stage musculoskeletal disorders [3, 4]. In order to prevent bleeding, perioperative intensive coagulation factor replacement treatment to increase plasma levels of FVIII is inevitable, especially for major orthopaedic procedure leading to a peak treatment moment of FVIII and a higher dose compared with a spontaneous bleed [5].

The development of factor VIII inhibitor antibodies is the most important complication of the treatment of HA, as it renders the administered FVIII concentrates ineffective, leading to increased complications and mortality [6, 7]. In literature, intensive treatment with factor VIII concentration for surgical procedure in HA has been proposed to be associated with higher FVIII inhibitor development, which leads to increased postoperative surgery related and hematologic complications and mortality because of the ineffective FVIII administration [8]. According to a meta-analysis, the overall prevalence of inhibitors in unselected haemophiliac populations was found to be 5–7%, and the reported crude incidence varied from 0 to 33% [9]. It is important to know the incidence of inhibitor development in people with HA undergoing major orthopaedic surgery, because this risk has to be considered and well-informed when an elective surgical procedure is planned. The purpose of this retrospective study is to analysis the incidence of inhibitor development in a consecutive group of previous treated HA people (PTPs) receiving intensive FVIII replacement therapy for major orthopaedic surgical procedures.

## Materials and methods

### Patient selection

The study was approved by the Institutional Review Board of Peking Union Medical College Hospital. The medical records of PWHA undergoing orthopaedic surgical treatment between January 2002 and December 2018 in our institute were retrospectively reviewed. The inclusion criteria were defined as: (a) PTPs with more than 50 exposure days (EDs). (b) PWHA underwent major orthopaedic surgery. The major procedure in our study included total knee arthroplasty (TKA), total hip arthroplasty (THA), total elbow arthroplasty (TEA), ankle arthrodesis (AA), surgery for haemophilia complicated with fracture, surgery for haemophilia complicated with pseudotumor. (c) PWHA accepted perioperative intensive replacement treatment of FVIII. The perioperative intensive treatment was defined as the cumulative use of at least 10,000 iu or 250 iu/kg for 5 or more consecutive days [10]. Patients with a known past history of a FVIII inhibitor or had any other haemostatic disorder were excluded from the analysis. Informed consent to participate was obtained from all the patients.

### Surgical procedures

Multiple joints procedures (MJP) during one anesthetic episode are considered when the patients suffer from multiple joints involvement to reduce the event of intensive treatment with factor concentration [11]. All the THAs were performed from posterior-lateral approach with cementless implants. All the TKAs were performed under tourniquet and the synovium was completely removed to reduce the recurrent haemarthroses and pain. AA was performed by intramedullary nailing with autograft or allograft. The surgical technique for haemophilic pseudotumor was introduced in the previous literature [12].

### Hematological care

A preliminary test of FVIII was conducted for all the cases before the operation for pharmacokinetic evaluation. The FVIII level and the factor inhibitor level were tested before the operation. Plasma derived FVIII or recombinant FVIII were used for HA. We referred to the guidelines of World Federation of Hemophilia to assign the strategy of clotting factor replacement therapy [5]. The patients were tested for inhibitors when the patients presented with ineffective treatment with FVIII, otherwise the patients were tested for inhibitors at the follow-up visit after surgery. Inhibitors were tested by the Nijmegen modification of the Bethesda assay [13]. One dose of tranexamic acid was used for HA at the start of operation. Pharmacologic anti-coagulation was not used for all the patients.

### Data collection

The clinical data were collected before surgery and included: age, body weight, level of plasma FVIII coagulant activity before surgery, history of previous intensive treatments for bleeding or surgery and choice of regimen and FVIII product, co-morbidity. The intensive treatment was defined as the cumulative use of at least 10,000 iu or 250 iu/kg for 5 or more consecutive days. Data recorded perioperatively included: type of surgery, type of FVIII concentrate, mode of FVIII administration, cumulative amount of FVIII concentrate administered. The patients were regularly followed at postoperative 2 weeks, 6 weeks, 3 months and annually thereafter. Any post-operative complications within postoperative 90 days exclusive of inhibitor development were recorded, considering that the complications were mainly related to the comorbidity of PWHA and perioperative management.

The primary outcome in this study was clinically relevant inhibitor development after surgery. A titre of 1–5 BU/ml was defined as a low inhibitor titre, and a titre of at least 5 BU/ml was defined as a high inhibitor titre [14]. The duration between the perioperative first exposure to factor and diagnosis of positive inhibitor was recorded.

### Statistical analysis

The clinical data was analyzed using means and standard deviation (SD). For categorical variables, chi-square analysis was used to compare the difference. The level of statistical significance was set at *P* < 0.05. To determine the independent predictors associated with development of positive FVIII inhibitor, univariate and multivariate logistic regression analyses were performed. Predictors factors for analysis included age ; presence of postoperative complication; level of baseline FVIII: C (< 1%, 1%~5%, > 5%); surgery information (total joint arthroplasty (TJA), AA, fracture, pseudotumor); choice of FVIII product; comorbidity of HIV or hepatitis infection; blood transfusion during surgery; operation strategy (single procedure or multiple procedures during one anesthesia. For regression analyses, the 95% confidence intervals (CIs) of odds ratios (ORs) were reported. Significant independent predictor variables were identified as those that maintained *P* values < 0.05 and an OR exclusive of 1.0. All statistical analyses were performed using SPSS 15.0 (SPSS, Inc., Chicago, IL, USA).

## Results

### Patient characteristics

A total of 193 haemophilia patients underwent 270 major orthopaedic surgery during the period. 32 patients with less than previous 50 EDs and 10 patients with haemophilia B were excluded. A total of 151 PTPs HA people who underwent 221 surgical procedures for haemophilic musculoskeletal disorders in our institution were included in this study. The demographic information was presented in Table 1. According to the baseline FVIII: C level, 111 patients were severe haemophilia with the average FVIII level of 0.41%±0.17%. 28 patients were moderate with the average level 2.1%±1.2%. 12 patients were mild with the average level of 17.2%±9.4% (Table 1). Plasma derived FVIII was used for 142 patients, and recombinant FVIII was used for 9 patients. Twenty patients (13%) accepted prophylaxis treatment with FVIII before, the rest of the patients accepted on demand of FVIII treatment. Thirty-seven patients (24.5%) had previous history of intensive FVIII treatment for surgical procedure (Table 1). Gene sequencing revealed a single-base duplication mutation *F8*:c.3637dupA(p.Ile1213Asnfs*28) in case5 and a small indel mutation *F8*:c.3635_3636delinsT(p.K1212Ifs*6) in case7.

**Table 1:** Demographic information and baseline characteristics of the type A haemophila patients who underwent major orthopaedic surgical procedures in this study

Item	Value (ratio)
Case number (n)	151
Age(years)	35.7 ± 9.8
BMI(Kg/m^2^)	23.42 ± 3.27
Body weight (Kg)	66.7 ± 12.5
Baseline FVIII: C Level	
<1%	111 (74%)
2-5%	28 (18%)
>5%	12 (8%)
HIV infection (n)	2
Hepatitis virus infection (n)	35
Previous history of intensive FVIII treatment for surgery (n)	
Yes	37 (24.5%)
No	114 (75.5%)
FVIII product	
Plasma-derived	142 (94%)
Recombinant	9 (6%)

n: case number; BMI: body mass index; HIV: human immunodeficiency virus; EDs: exposure days; FVIII: coagulation factor VIII;

### Surgical procedure

The surgery information was presented in Table 2. Sixty-two patients (41.1%) underwent multiple joints procedures (MJP) of total 132 surgical procedures during one anesthetic episode. Patients were exposed to FVIII concentrates for a median of 13.8 cumulative EDs (range 12 to 15) following surgery before stitch removal and received a mean perioperative cumulative FVIII of 498 iu/kg within first week after surgery and a mean cumulative FVIII of 157 iu/kg within the second week after surgery. In this group, except for inhibitor development, 27 patients (17.9%) totally experienced 29 postoperative complications within 90 days. The detail of the complication was presented in Table 2. The Hematologic complication included 9 cases of hemorrhage and 1 case of deep vein thrombosis.

**Table 2:** Surgical procedures information and complication within postoperative 90 days of the type A haemophila patients who underwent major orthopaedic surgical procedures

Item	*N* (ratio)
Case number (n)	151
Surgery strategy (n)	
Single procedure	89 (59%)
Multiple procedure	62(41%)
Total procedure information (n’)	221
TKA	120 (54.5%)
THA	57 (25.8%)
AA	15 (6.8%)
Pseudotumor	21 (9.5%)
Fracture	8 (3.6%)
EDs within postoperative two weeks(d)	13.7 ± 0.85
Cumulative dose of FVIII product (iu/Kg)	
POD0-POD7	498 ± 112
POD8-POD14	157 ± 73
Complication (n, incidence)	
Hematologic complication	10 (6.6%)
Wound complication	9 (5.9%)
Surgery related	10 (6.6%)
Blood transfusion (n)	69 (45.6%)

n: case number; n’: procedure number; TKA: total knee arthroplasty; THA: total hip arthroplasty; AA: ankle arthrodesis; EDs: exposure days; FVIII: coagulation factor VIII; POD: postoperative day

### Inhibitor development

Seven cases (4.6%) developed an inhibitor post-operatively in our study. The detail of the cases with FVIII inhibitor development was presented in Table 3. The duration between the perioperative first exposure to factor and diagnosis of positive inhibitor was from POD3 to 5 years. Six of the patients developed positive inhibitor within 30 days after operation (average 12 days, range 3 to 30). The other one case (case 5) were diagnosed of positive inhibitor at five years later (Table 3). He was diagnosed until the second admission for complication of pseudotumor and pathological fracture. Two cases (case 3 and case 7) had the transient inhibitor within six months. The average peak level of inhibitor in this study was 17.8 BU/ml (rang, 1.2 to 64 BU/ml). In total, 6 (85.7%) cases were diagnosed as severe haemophilia according to baseline FVIII level, 4 (57.1%) cases had a high titer inhibitor development after surgery, 6 (85.7%) cases had history of intensive FVIII exposure for previous surgery, 2 (28.6%) cases underwent surgery for haemophilia pseudotumor (Table 3). Five of the seven cases experienced postoperative complications except for inhibitor development within postoperative 90 days, with the rate of 71%, which was higher than the complication rate of 17.5% in the entire group.

**Table 3:** Detailed information of the Type A haemophila patients who developed FVIII inhibitor after major orthopaedic surgical procedures in this study

Case No.	Age	Body weight(Kg)	Baseline FVIII: C level	Mutation	Year of surgery	Diagnosis	Surgery	FVIII product	Description of the case	Peak level of inhibitor	Previous history of intensive FVIII treatment
Case 1	26	57	1.2%	NA	2005	Right femur pseudotumor with pathologic fracture	Pseudotumor resection and ORIF	Plasma-derived	Hematoma at POD8 of surgery with positive inhibitor. Symptom relieved after treatment with PCC.	21BU/ml	Underwent surgery for right femur shaft fracture in 2003.
Case 2	47	80	2.1%	NA	2009	Left femur pseudotumor with infection	Pseudotumor resection, Debridement	Plasma-derived	Increase of drain and local hematoma at POD3 after pseudotumor resection. Positive inhibitor was developed. Symptom relieved and the patient recovered with the administration of recombinant factor VIIa (rFVIIa).	2 BU/ml	Underwent surgery for left femur Pseudotumor complicated with fracture in 1996 in our institute. History of local trauma and infection 2 months ago.
Case 3	25	50	1.1%	NA	2010	Right knee arthropathy	TKA	Plasma-derived	Hemarthrosis at POD7 of TKA with positive FVIII inhibitor. Symptom relieved after treatment with PCC.	1.2 BU/ml	Left TKA in 2007.
Case 4	41	75	0.2%	NA	2014	Right ankle arthropathy	AA	Recombinant	Positive FVIII inhibitor at 30 days after right AA.	16 BU/ml	History of bilateral TKA in 2013.
Case 5	48	61	0.1%	*F8*: c.3637dupA(p.Ile1213Asnfs28)	2013	Bilateral hip arthropathy	THA	Plasma-derived	Underwent bilateral THAs in 2013。Positive FVIII inhibitor developed in 2018, complicated with pseudotumor and pathologic fracture.	35.2 BU/ml	Yes
Case 6	29	54.5	0.1%	NA	2016	Left knee arthropathy	TKA	Plasma-derived	Positive FVIII inhibitor at 15 days after left TKA.	16 BU/ml	NA
Case 7	32	56	0.2%	*F8*:c.3635_3636delinsT(p.K1212Ifs6)	2018	Left knee arthopathy	TKA	Plasma-derived	Positive FVIII inhibitor at POD10 of left TKA.	4.8 BU/ml	Right TKA in 2014.

TKA: total knee arthroplasty; THA: total hip arthroplasty; AA: ankle arthrodesis; FVIII: coagulation factor VIII; ORIF: open reduction and internal fixation; POD: postoperative day; PCC: prothrombin complex concentrate

Surgical procedure for pseudotumor had the higher incidence of inhibitor development compared with TJA (9.5% vs. 3.5%, χ^2^ = 1.53, *P* = 0.215). The group of patients experienced postoperative complications had the higher incidence of inhibitor development compared with the patients without postoperative complication (13.3% vs. 2.4%, χ^2^ = 6.55, *P* = 0.01). There was no statistically significant difference of the inhibitor development between MJP and single joint procedure (1.6% vs. 6.7%, χ^2^ = 2.174, *P* = 0.14), as well as between severe haemophilia and mild/moderate haemophilia (3.6% vs. 7.5%, χ^2^ = 1.01, *P* = 0.315). Multivariate logistic regression analysis was further used to identify risk factor for the complication of inhibitor development. Only previous history for intensive FVIII exposure was considered as a significant predictor for postoperative inhibitor development (*OR*: 29.5, *P* = 0.002)(Table 4).

**Table 4:** Result of multivariate logistic regression analysis for the possible risk factors of positive FVIII inhibitor after major orthopaedic surgical procedures in type A haemophiliac patients

Item	*P*-value	OR (95% CI)
Previous history for intensive FVIII	0.002	29.5 (3.5 ∼ 246)
Postoperative complication	0.285	-
Blood transfusion (n)	0.918	-
Comorbidity of HBV/HCV/HIV	0.128	-
Age(years old)		
<20	-	
20 ∼ 50	0.105	-
>50	0.156	-
Procedure		
TJA	-	
AA	0.358	-
Pseudotumor	0.493	-
Fracture	0.444	-
Baseline FVIII: C Level		
<1%	-	
2-5%	0.421	-
>5%	0.485	-
Multiple procedure vs. single procedure	0.349	
FVIII product	0.358	-

TJA: total joint arthroplasty; AA: ankle arthrodesis; FVIII: coagulation factor VIII; EDs: exposure days; HBV: Hepatitis B virus; HCV: Hepatitis C virus; HIV: human immunodeficiency virus; CI: confidence interval

## Discussion

Perioperative FVIII replacement regimens are targeted to prevent bleeding, and has resulted in a tendency to aim for higher FVIII levels, which leading to the use of higher doses of factor concentrates in surgical procedures [15]. The major orthopaedic surgical procedure consists of more invasive manipulation and postoperative rehabilitation. So more intensive factor treatment is required for the major orthopaedic surgical procedure [5]. There are numerous reports about the incidence of inhibitor development in haemophilia patients and a small ratio of the patient undergoing surgical procedure were included in these reports [8–10, 16, 17]. However, the most performed procedures were dental surgery and catheter implantation, limited study focused on the incidence of inhibitor in major orthopaedic procedure [18]. 

In literature, inhibitor development is associated with haemophilia patients’ genotype and might be triggered by environmental factors during their treatment, such as intensive treatment with clotting factor, inflammation and infection [19, 20]. Inflammation may provoke antibody formation in B lymphocytes by the concurrent presence of so called ‘danger signals’ of cytokine release arising from injured tissues [21, 22]. Probably, surgical procedure may make patients prone to inhibitor development by causing tissue damage and inflammation.

This is one of the first studies focusing on the incidence of inhibitor development after intensive FVIII treatment for major orthopaedic surgery in PWHA. Seven cases (4.6%) developed inhibitor post-operatively in our study. The incidence of inhibitor development after major orthopaedic surgery in this cohort of consecutive patients was lower than the results of inhibitor development after intensive treatment in literature [23–25]. Gouw et al. [23] reported the overall cumulative incidence of inhibitors was 32.0% in 576 children with severe HA accepting intensive FVIII treatment. Gouw et al. [24] reported another group of 366 severe previous untreated PWHA. Eighty-four patients accepted surgical procedures under replacement therapy at least three consecutive days. Eighty-seven (24%) patients developed inhibitor, and the patients who were first treated for surgical procedures had a markedly higher risk of inhibitor development (65%) than patients who were first treated for bleeding (23%). Eckhardt et al. [25] reported a retrospective cohort study of 138 moderate/mild haemophilia patients, found an inhibitor incidence of 17% after surgery (7/41). One of reason of the higher inhibitor incidence in those studies may be explained by the selection of high-risk patients for inclusion. Gouw et al. [23] reported the inhibitor incidence in children with average age of 9.8 months within their first 75 FVIII exposure days. Gouw et al. [24] and Eckhardt et al. [25] reported another groups of previously untreated patients. All these factors may increase the risk of inhibitor development [17, 18].

According to the report in literature, the higher number of EDs prior to surgery always led to less inhibitor development after factor treatment [9, 18, 24]. In the present study, we only included the patients with more than 50 EDs and all the patients were admitted for end-stage arthropathy and/or musculoskeletal disorders, which meant there was a long history of the disease before admission. Furthermore, the age of the patients ranged from 16 to 61 years old in this study, which was not peak period for inhibitor development throughout the life [17]. The rate of inhibitor development in this study was consistent with the result in some literatures [24, 26, 27]. We predicted the aforementioned points, as well as some others predisposing factor such as ethnicity, regimen of FVIII treatment, might be the reason of low incidence of inhibitor development in this study.

In this study, 4 (57.1%) cases had a high titer inhibitor development after surgery. The result was consistent with the results in literature [14, 17, 23]. Gouw et al. [23] reported the ratio of high titer inhibitor over all the inhibitor cases in severe PWHA accepting intensive FVIII treatment was 65% (118/179). Van et al. [14] reported 39 cases of 75 positive inhibitor patients had high titer inhibitor after intensive FVIII treatment and the median inhibitor peak titer was 7 BU/mL (IQR, 2–26). The interesting result of this study was that the mean peak titer of inhibitor was 22.6 BU/mL (1.2 to 64 BU/ml), which was higher than the results in literature. The high level of the inhibitor might be related to the characteristics of more invasive orthopaedic procedure [15], and would inevitably increase the further treatment burden [28, 29].

In this study, surgical procedure for pseudotumor had the higher incidence of inhibitor development than TJA. The patient experienced postoperative complication also had higher incidence of inhibitor development. We concluded the reason might be related to the more tissue damage and inflammation during the surgical procedure, as well as during the presence of postoperative complications [9, 11, 18].

There were several studies about the treatment-related risk factors of inhibitor development in patients with haemophilia in literature [8, 10, 14, 18, 24, 30, 31]. The brief review of the studies about intensive FVIII treatment and inhibitor development in HA was summarized in Table 5 [10, 14, 23–25, 32–35]. However, the included patients consisted of a wide crowd and unselected haemophiliac populations, including previous untreated patients [23–25], patients accepted prophylaxis treatment [17, 31], patients accepted intensive treatment for bleeding, patients accepted any surgery procedure [24], et al. Most of the studies concluded that high-dosed intensive FVIII treatment for surgery would increase the inhibitor risk (see Table 5). In this study, we concentrated on the haemophiliac patients undergoing major orthopaedic surgery, and tried to identify, by multivariate logistic regression analysis, the risk factor for the complication of inhibitor development. After adjustment for all measured potential confounding factors, association between the observed factors and risk of inhibitor development that was present in the previous part largely disappeared (see Table 4) [36]. Only history of previous intensive FVIII exposure was considered as a statistically significant predictor for postoperative inhibitor development. The reason may be explained by the immunological ‘danger theory’ of P. Matzinger [21, 22]. Previous surgery under intensive FVIII treatment made the patients presence of ‘danger signals’, which confers an increased risk of inhibitor development after additional administration of FVIII. Our result was consistent with one of the latest studies in literature [14], which reported the ever previous surgery rendered non-severe haemophila A patients 4.2 fold risk of inhibitor development after intensive factor treatment.

**Table 5:** Summary of the literature studies on intensive FVIII treatment, surgical procedure and inhibitor development in HA

Author, year [reference]	Study design	Patients(*n*)/Severity of disease	Previous FVIII exposure	All surgery information (*n*)	Inhibitor development (*n* /%.)	Total surgeryes(*n*)/ inhibitor(*n*)	Risk factor (RR, OR)
Gouw et al. 2007 [24]	R	366 Severe	366 PUP	63 portacath implantations 21 other surgery	87(24%)	84/NS	Surgery (RR, 3.7) early intensive treatment (RR, 3.3)
Gouw et al.2007 [32]	R	272 severe	36 PTP	44 all surgery	67(28%)	44/NS	Intensive treatment periods (RR, 1.6) surgery (RR, 2.7)
Eckhardt et al. 2009 [25]	R	128 mild, 10 moderate	138 PUP	41 all surgery	10(7%)	75/7	The Arg593Cys (RR,10) intensive peri-operative use of FVIII (RR, 186)
Eckhardt et al.2012 [10]	*P*	43 mild, 3 moderate	13 PTP	30 all surgery 12 orthopaedic surgery	2(4%)	46/2	NS
Gouw et al. 2013 [23]	*P*	576 non-severe	576 PUP	144 major surgery	179(32%)	NS	High-dose FVIII treatment (HR, 2.3)
van Velzen et al. 2017 [14]	CC	298 mild/moderate 75 with inhibitor 223 without inhibitor	NS	NS	NS	NS	Surgical intervention (OR 4.2) mean dose > 45 IU·kg^−1^·ED^−1^ (OR 7.5)
Osooli et al. [33] 2017	R	167 severe	NS	32 joint surgery	26(21.5%)	NS	NS
Iorio et al. [27] 2017	C	4443 severe	All PTP	NS	29(0.65%)	NS	Surgery; high intensity treatment periods
Tong et al. [34] 2018	R	77 severe 68 non-severe	NS	49 major procedure 14 orthopaedic	0%	-	NS
Kim et al. 2019 [35] 2019	R	89 severe 21 moderate 5 mild	NS	NS	10(8.7%)	NS	Surgery Short-term large exposure

n: case number; P: prospective; R: retrospective; C: case series; CC: case control; NS: not stated; PUP: previous untreated patient; PTP: previously treated patients; RR: relative risk; OR: odds ratio; HR: hazard ratio; ED: exposure day

We got the Sanger-sequencing result of case 5 (see Table 3) by himself and the report showed that he carries a single-base duplication mutation *F8*: c.3637dupA(p.Ile1213Asnfs*28). Then third-generation sequencing were performed to detect the pathogenic mutation in case 7 (see Table 3) by Berry Genomics Corporation (Beijing, China) and found a small indel mutation c.3635_3636delinsT(p.K1212Ifs*6) in *F8* gene. Interestingly, these two mutations were very closely located in poly A regions of *F8* B domain and all has been reported in inhibitor negative severe HA cases before [37]. Meanwhile, it was reported that patients with large deletions, nonsense mutations and intron 22 inversions had a 7–10 fold higher risk of developing inhibitors than patients with small indels and point mutations and the risk of developing inhibitors was higher in patients with mutations in the A3 and C2 domains compared to mutations in the B domains [38, 39]. This may suggest that PWHA have small indels in polyA regions of *F8* B domain have a low risk of inhibitor developments, but would be provoked to inhibitor positive after intensive FVIII treatment for major orthopaedic surgical procedures.

While the major strength of our study is the focus on the inhibitor development after intensive FVIII treatment for major orthopaedic surgical procedure, the present study still has several limitations. First, this study was a retrospective study, which risks low data homogeneity and integrity compared with prospective study. For example, all the FVIII was administered by intermittent bolus injections in this study, so we could not analysis the effect of continuous infusion to the inhibitor development. Secondly, because of the limited case number, this study might be underpowered to detect the potential difference of inhibitor development between severe haemophilia and mild/moderate haemophilia. Multicenter studies with more case number are needed in future. Thirdly, the etiology of inhibitor production might be genetic. We did not detect all patients’ *F8* mutations in this study, which implied we might miss some genetic risk factor of inhibitor development. Fourthly, undetected low-titer inhibitors were found in up to 8% of patients according to literature [40]. According to this study, we advocated multiple procedures under one anesthetic episode for haemophilia patients in order to avoid multiple admissions to decrease the risk of inhibitor development [11].

## Conclusion

In conclusion, the average inhibitor incidence of intensive FVIII treatment for major orthopaedic surgical procedure was 4.6% in this study. Our findings suggest the history of previous intensive FVIII treatment for surgery was significant risk factor for inhibitor development in PWHA accepting intensive FVIII treatment for major orthopaedic surgical procedure. The surgical procedure for haemophilic pseudotumor, the patient experienced postoperative complication can also increase the incidence of inhibitor development. The surgeon should be aware of inhibitor risk in these patients and be well-informed when an elective surgical procedure is planned.

## Data Availability

No datasets were generated or analysed during the current study.
